# Vitamin C in Cardiovascular Disease: From Molecular Mechanisms to Clinical Evidence and Therapeutic Applications

**DOI:** 10.3390/antiox14050506

**Published:** 2025-04-23

**Authors:** Yichen Xu, Huabo Zheng, Ioana Slabu, Elisa Anamaria Liehn, Mihaela Rusu

**Affiliations:** 1 Key Laboratory of Tropical Translational Medicine of Ministry of Education, School of Basic Medicine and Life Sciences, Hainan Medical University, Haikou 571199, China; yichenxu@muhn.edu.cn; 2 Department of Cardiology, Fujian Medical University Union Hospital, Fuzhou 350001, China; 3 Institute of Applied Medical Engineering, Helmholtz Institute, Medical Faculty, RWTH Aachen University, 52074 Aachen, Germany; 4 Department of Electrical Engineering, Helmut Schmidt University, 22043 Hamburg, Germany; 5 National Heart Center Singapore, 5 Hospital Dr., Singapore 169609, Singapore; 6 Center for Innovation and eHealth, University of Medicine and Pharmacy Carol Davila, Pitar Mos 20, 010451 Bucharest, Romania

**Keywords:** vitamin C, antioxidant, cardiovascular diseases, myocardial infarction, collagen synthesis, ECM remodeling

## Abstract

Vitamin C, also known as ascorbic acid, is an essential nutrient that humans cannot synthesize, making its intake crucial for health. Discovered nearly a century ago, vitamin C is widely recognized for its ability to prevent scurvy and has become one of the most commonly used supplements. Beyond its antioxidant activity, vitamin C is pivotal in regulating lipid metabolism, promoting angiogenesis, enhancing collagen synthesis, modulating remodeling, and stabilizing the extracellular matrix. While preclinical studies have shown promising results, clinical trials have yielded inconsistent findings, due to suboptimal study design, results misinterpretation, and misleading conclusions. This review provides a holistic overview of existing evidence on the pleiotropic role of vitamin C in cardiovascular diseases, identifying both the strengths and limitations of current research and highlighting gaps in understandings in vitamin C’s underlying mechanisms. By integrating molecular insights with clinical data and evaluating the pleiotropic role of vitamin C in cardiovascular disease management and prevention, this review aims to guide future research toward personalized, evidence-based therapeutic strategies in clinical practice.

## 1. Introduction

Vitamin C, also known as ascorbic acid (the anti-scurvy vitamin), is an essential human body nutrient, first identified in the 1920s by Albert von Szent Györgyi [1]. Unlike complex vitamins B [2] and D [3,4], the human body cannot produce vitamin C [5]. Over the 20th century, vitamin C became the most widely used supplement in recorded human history. Beyond its well-known role in preventing scurvy [6], vitamin C has pleiotropic biological effects [7,8], contributing to various physiological functions throughout the body. A deeper understanding of its multifaceted roles is essential particularly for cardiovascular health, owing to the loss of its biosynthesis in humans.

Cardiovascular diseases (CVDs), such as myocardial infarction (MI) and atherosclerosis (AS), continue to represent major global health challenges [9]. These conditions involve complex mechanisms, including oxidative stress, endothelial dysfunction, acute and chronic inflammation, extracellular matrix (ECM) remodeling, and fibrosis [10]. Collagen, a key component of the cardiac ECM and fibrotic tissue, plays a pivotal role in maintaining structural integrity and supporting the cellular processes necessary for tissue repair [11,12]. Ensuring an optimum balance between collagen synthesis and degradation is critical for the homeostasis of healthy heart function effective pathological cardiac remodeling as any imbalance can significantly impair the heart’s biomechanical properties and its capacity for regeneration [13,14].

These pleiotropic effects—ranging from antioxidant and anti-inflammatory actions to extracellular matrix regulation—make vitamin C a potential therapeutic candidate for cardiovascular diseases [15,16]. Despite promising preclinical data, clinical trials examining vitamin C’s effects on cardiovascular disease health have produced inconsistent results [15,17]. Understanding the reasons behind these discrepancies is crucial for optimizing its therapeutic potential in clinical settings.

While its role in anti-oxidation and inflammation is extensively studied, the additional roles of vitamin C in angiogenesis, extracellular matrix remodeling, and fibrosis are underscored for considering vitamin C as a therapeutic agent for cardiovascular diseases. Our review aims to provide a holistic overview that goes beyond the current state-of-the-art, examining molecular mechanisms as well as preclinical and clinical factors influencing vitamin C’s efficacy in cardiovascular diseases. By critically evaluating existing literature, the review seeks to identify both the strengths and limitations of current research, highlight gaps in understanding, and propose areas where further investigation is needed. The goal is to develop a deeper understanding of how vitamin C can be effectively utilized in cardiovascular care and to lay the groundwork for evidence-based guidelines that will optimize its therapeutic use in clinical practice and outlining future research directions.

## 2. Vitamin C Deficiency and Human Health

The inability of humans to synthesize vitamin C increases susceptibility to oxidative stress, hypertension, and atherosclerosis, as demonstrated in numerous studies. Vitamin C synthesis, absorption, accumulation, and excretion follow a complex biochemical pathway converting glucose into glucuronic acid, a key metabolite for detoxification and metabolic regulation. The following sections will discuss these processes in detail. The efficient transport of vitamin C is facilitated by specialized transporters and diffusion mechanisms, which are critical for maintaining antioxidant defenses and cellular health. Vitamin C levels can be enhanced through dietary intake or supplementation, both of which help reduce oxidative stress and inflammation, supporting cardiovascular health. A balanced approach combining dietary intake with targeted supplementation ensures optimal vitamin C levels, addressing both general health needs and specific health conditions.

### 2.1. Evolutionary Loss and Cardiovascular Implications

In most plants and animals, vitamin C synthesis involves a complex biochemical pathway with multiple steps converting glucose into vitamin C. A pivotal stage in this process is the enzymatic conversion of glucose to glucuronic acid, which has a crucial biochemical role in the detoxification and regulation of various metabolic processes in the body [18,19]. In mammals capable of synthesizing vitamin C, key enzymes, such as UDP-glucose dehydrogenase, SMP30 (regucalcin), and L-gulono-1,4-lactone oxidase (GULO), are essential in the biosynthetic pathway, each facilitating specific steps in the conversion of glucose to ascorbic acid [20,21]. However, humans, along with some primates and fruit bats, deviate from this pattern due to mutations that inactivated the GULO gene over evolutionary time [22].

**Figure 1:**
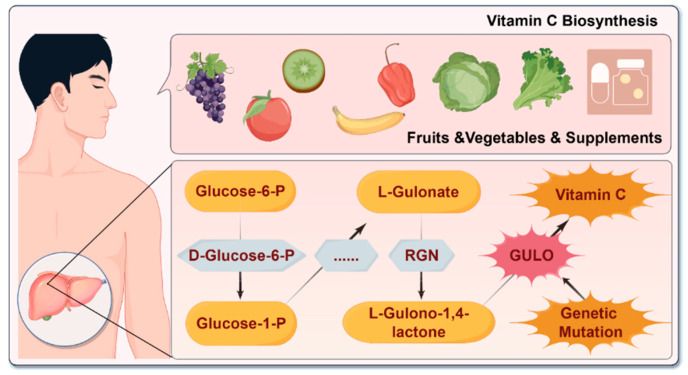
Schematic overview of the vitamin C biosynthetic pathway from glucose-6-phosphate to ascorbic acid. In species capable of vitamin C synthesis, glucose-6-phosphate (Glucose-6-P) is enzymatically converted into glucose-1-phosphate (Glucose-1-P), followed by its transformation into L-gluconate and subsequently L-gulono-1,4-lactone. This final intermediate is oxidized to produce vitamin C (ascorbic acid) via the enzyme L-gulono-1,4-lactone oxidase (GULO), with regulatory support from regucalcin (RGN). In humans, genetic mutations have rendered GULO non-functional, thereby halting the final step and making the dietary intake of vitamin C essential. **Abbreviations:** Glucose-6-P: glucose-6-phosphate; Glucose-1-P: glucose-1-phosphate; RGN: regucalcin; GULO: L-gulono-1,4-lactone oxidase.

Humans cannot synthesize vitamin C due to the lack of key biosynthetic enzyme GULO (Figure 1). It has been proven that the inactivation of the *GULO* gene was probably due to a series of mutations that occurred over evolutionary time [18]. These mutations, such as single nucleotide deletions, single nucleotide insertions, and trinucleotide deletions, likely rendered the *GULO* gene non-functional, resulting in the loss of the ability for vitamin C synthesis [19]. Factors such as genetic variability, dietary habits, and health conditions influence both the synthesis and absorption of vitamin C, which can significantly affect disease susceptibility, progression, and prognosis.

Studies on *GULO*^-/-^ mice have demonstrated the severe health consequences of vitamin C deficiency, including impaired collagen synthesis, increased oxidative stress, and heightened susceptibility to cardiovascular diseases [23,24]. For example, *GULO*^-/-^ mice develop hypertension, atherosclerosis, and endothelial dysfunction, among other pathologies [25], highlighting the essential role of vitamin C in maintaining cardiovascular health and overall physiological stability, which are highlighted in Table 1. These findings underscore the critical importance of vitamin C not only in metabolic processes but also in disease prevention and health maintenance.

Although both studies utilized *GULO*^-/-^ C57BL/6 mice, the pathological outcomes differed significantly due to variations in experimental conditions. In Kim et al. [26], mice underwent abrupt vitamin C withdrawal combined with physical restraint stress, leading to acute cardiovascular injury, including endothelial dysfunction, aneurysm formation, and increased mortality within 2 weeks. In contrast, Cha et al. [24] maintained mice under vitamin C-deficient conditions without external stressors, and observed progressive vascular dysfunction and lipid profile alterations over a longer period. These differences suggest that external stress and experimental duration can markedly influence the severity of cardiovascular phenotypes in *GULO*^-/-^ models.

### 2.2. Absorption, Tissue Accumulation, and Excretion of Vitamin C

The primary site for vitamin C absorption in humans is the small intestinal epithelium, where sodium-dependent vitamin C transporters (SVCTs) facilitate uptake [28]. SVCT1 transports and facilitates the absorption of vitamin C under physiological conditions [29] by involving its conformational changes and the cooperative transport of sodium ions (Na^+^) [30]. For instance, the binding of sodium ions at the Na1 site, coordinated by Glu341, Asp345, Ser390, and vitamin C itself, plays a critical role in neutralizing the local negative charges [31]. The initial Na^+^ binding increases SVCT1’s affinity for vitamin C, while a second Na^+^ binding stabilizes the complex and enhances transport efficiency. This dual Na^+^ binding mechanism underscores the intricacy of SVCT1-mediated vitamin C transport and highlights the importance of electrolyte balance in the effective absorption and utilization of vitamin C.

**Figure 2:**
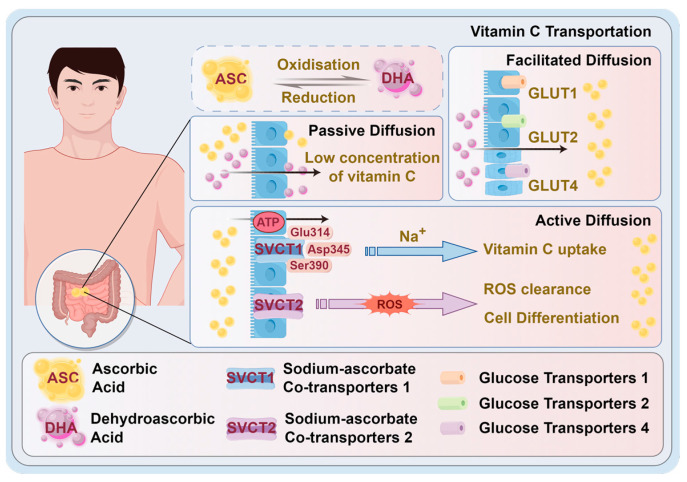
Schematic representation of vitamin C transport mechanisms in the human body. Vitamin C (ascorbic acid) is actively transported into cells via sodium-dependent vitamin C transporters SVCT1 and SVCT2, with SVCT1 mediating intestinal absorption and SVCT2 distributing ascorbate to metabolically active tissues. In contrast, dehydroascorbic acid (DHA), the oxidized form of vitamin C, enters cells via facilitated diffusion through glucose transporters (GLUT1, GLUT2, GLUT4). Once inside, DHA is reduced back to ascorbate by intracellular antioxidants. **Abbreviations:** SVCT: sodium-dependent vitamin C transporter; DHA: dehydroascorbic acid; GLUT: glucose transporter.

SVCT2 is primarily localized to the basolateral surface of epithelial cells, where it mediates the uptake of vitamin C from the bloodstream into cells, supporting tissue-specific accumulation and intracellular antioxidant protection [32] (Figure 2). This transporter is vital for tissues with high metabolic demand, playing a key role in regulating Reactive oxygen species (ROS) levels and driving cellular differentiation [33,34]. When vitamin C participates in enzymatic reactions inside cells, it is converted into dehydroascorbic acid. In contrast to vitamin C, dehydroascorbic acid can passively diffuse through cell membranes in the presence of sugar transporter isoforms, like GLUT1, GLUT2, and GLUT4 [35]. Inside the cells, dehydroascorbic acid is rapidly reduced back to vitamin C by glutathione, thioredoxin, and nicotinamide adenine dinucleotide phosphate (NADPH) [36]. This recycling mechanism maximizes the utilization of available vitamin C and is crucial for maintaining redox balance; its dysfunction can contribute to the pathogenesis of cardiovascular diseases. Ascorbic acid and dehydroascorbic acid are excreted by kidneys when the renal threshold is exceeded. At low intake of vitamin C, kidneys reabsorb vitamin C, resulting in slower excretion. However, at high intake levels, excess dehydroascorbic acid, which is not converted back to vitamin C is broken down into oxalic acid and oxalates, which are main contributors to kidney stone formation [37].

As an additional consideration, the influence of ethnicity on vitamin C metabolism and efficacy is essential to address. Considering the influence of ethnicity on vitamin C metabolism and efficacy is essential. Although most clinical evidence has been derived from caucasian populations, emerging research indicates that genetic, dietary, and physiological differences across ethnic groups may significantly affect vitamin C absorption, plasma concentrations, recommended dietary allowances (RDA), and clinical responsiveness [38]. These factors can lead to different vitamin C requirements across racial groups, thereby affecting global dietary recommendations for vitamin C.

For instance, recent data from a large NHANES-based cross-sectional study found that the average dietary vitamin C intake among U.S. adults included in the study was 82.5 mg/day, highlighting that a substantial proportion of the population may not meet optimal intake levels through diet alone [39]. Similarly, a nationwide cross-sectional study among Chinese adults reported an average dietary vitamin C intake of only 78.1 mg/day, with 65.1% of participants at risk of inadequate intake [40]. These findings highlight the significant variability in dietary patterns and nutritional risks across populations, underscoring the critical need for further research involving ethnically diverse cohorts. Such insights underscore the need to refine dietary recommendations and design targeted supplementation strategies tailored to different populations.

Together, these mechanisms of absorption, recycling, and excretion underscore the central role of vitamin C in maintaining systemic redox balance, preventing cardiovascular injury, and promoting physiological resilience across populations.

### 2.3. Supplement Forms and Clinical Implications

Vitamin C supplements are available in a variety of formulations, including tablets, capsules, powders, liquids, and liposomal preparations. These supplements may be derived from natural sources, including: citrus fruit, acerola cherries, and chemically synthesized via industrial processes [41]. Although the molecular structure of ascorbic acid is identical regardless of its origin, differences in purity, cofactor content, and bioavailability may exist between natural and synthetic forms [42].

The delivery format of vitamin C significantly affects its absorption efficiency and pharmacokinetics. For instance, liposomal and other encapsulated preparations have demonstrated improved stability and enhanced cellular uptake compared to non-encapsulated forms [43]. Recent advances in vitamin C nano-delivery systems, such as polymer micelles and gold nanoparticle conjugates, demonstrate enhanced chemical stability, glucose-responsive release, and effective cellular uptake, enabling controlled delivery across a wider concentration range with preserved antioxidant activity [44].

While vitamin C from whole foods is generally well absorbed under physiological conditions, controlled supplementation using synthetic, pharmaceutical-grade vitamin C can provide more predictable and sustained plasma levels, especially at therapeutic doses required for modulating inflammation, enhancing endothelial function, or supporting post-injury collagen remodeling [45]. The absence of plant-derived cofactors in synthetic preparations may limit synergistic antioxidant effects, but it also allows for standardized dosing and cleaner pharmacokinetic profiling, which are critical advantages for clinical application.

Recent clinical data have shown that intravenous administration of vitamin C (20 mg/kg during the rewarming phase of cardiopulmonary bypass) significantly improves erythrocyte deformability and reduces oxidative stress in patients undergoing cardiac surgery [46]. Ren et al. found that high-dose vitamin C supplementation (2000 mg/day for 7 days) significantly reduced biomarkers of inflammation and oxidative stress, such as interleukin-6 (IL-6) by 19.47%, tumor necrosis factor-alpha (TNF-α) by 17.30%, C-reactive protein (CRP) by 34.01%, systolic blood pressure by 3.37%, and pulse pressure by 6.03%, while simultaneously increasing glutathione peroxidase (GSH-Px) levels by 7.15% [47]. These findings underscore the role of high-dose vitamin C in mitigating cardiovascular risk factors and improving outcomes in individuals exposed to high levels of environmental pollutants.

The combination of vitamin C with other antioxidants, like vitamin E, holds considerable promise for enhancing cardiac regeneration and improving clinical outcomes in patients with CVD. For instance, vitamin C plays a key role in regenerating oxidized vitamin E, thereby sustaining its antioxidant function within lipid membranes [48]. This synergistic interaction suggests that combined supplementation of vitamins C and E may have amplified protective effects, particularly in reducing lipid peroxidation and improving endothelial function. A case-control clinic trial in CVD patients demonstrated that combined supplementation of vitamin C (500 mg/day) and vitamin E (400 IU/day) for two months significantly enhanced both enzymatic and non-enzymatic antioxidant defenses, while reducing lipid peroxidation by 40%, suggesting a synergistic effect in reducing oxidative stress [49].

Therefore, while both dietary sources and supplements of vitamin C exhibit their benefits, the natural dietary intake is generally preferred over synthetic supplementation for maintaining baseline levels and ensuring a continuous supply of this essential nutrient. However, in cases of increased need or specific cardiovascular conditions, further vitamin C supplementation can provide a necessary dose that is not achievable through diet alone.

## 3. Molecular Mechanisms of Vitamin C in Cardiovascular Regulation

Beyond its well-known antioxidant capacity, vitamin C is involved in multiple physiological processes critical to cardiovascular integrity, including modulation of inflammatory responses, preservation of endothelial function, and regulation of collagen remodeling. These mechanisms will be discussed in detail in the sections below.

### 3.1. Anti-Inflammatory and Endothelial Effects

Preclinical studies have shown that vitamin C influences several key molecular pathways, including NLRP3/caspase-1 [50], TLR4/MyD88/NF-κB [51], Nrf2/HO-1 [52], and MAPK [53]. These pathways collectively regulate inflammation, oxidative stress, and angiogenesis, all of which are critical in CVD progression. Despite the fact that the main role of vitamin C as a potent antioxidant is well-established [54], its clinical benefits in cardiovascular disease remain controversial [55,56]. This might be caused by the multiple and complementary beneficial effects of vitamin C on different pathology phases, which are currently underestimated or even ignored in clinical applications. One of vitamin C’s most underappreciated roles is its potent anti-inflammatory effect, achieved through the regulation of immune cell function and cytokine production [57,58]. For example, a clinical cohort study from NHANES 2017–2018 showed an inverse relationship between plasma vitamin C levels and high-sensitivity CRP (hs-CRP), a key marker of inflammation, with the most significant CRP reduction observed at plasma vitamin C levels up to 53.1 μmol/L [59]. Since elevated hs-CRP is linked to an increased risk of CVD, this finding suggests that vitamin C may offer cardioprotective effects by reducing inflammation. Additionally, in patients with gout [60] and those undergoing myeloablative chemotherapy [61], lower vitamin C levels correlate with higher hs-CRP, indicating that vitamin C deficiency may exacerbate inflammation. These findings, alongside its antioxidant properties, reinforce vitamin C’s potential in reducing cardiovascular disease risk through the modulation of inflammatory pathways.

Vitamin C has been shown to modulate IL-6 levels, a proinflammatory cytokine often elevated in both acute inflammatory events, such as acute coronary syndromes, and chronic inflammatory conditions, like CVD, rheumatoid arthritis, and metabolic disorders [62]. For example, a randomized controlled trial showed that daily supplementation of 1000 mg vitamin C significantly reduced IL-6 levels [63]. Furthermore, vitamin C’s antioxidant properties are underscored by its effect on malondialdehyde (MDA), a byproduct of lipid peroxidation recognized as a biomarker of oxidative stress and cellular damage [64]. This systematic review demonstrated that regular supplementation with 1000 mg of vitamin C significantly reduces MDA levels, highlighting vitamin C’s potent antioxidant capability. By neutralizing ROS and preventing lipid peroxidation, vitamin C protects cell membranes from oxidative damage. This reduction in MDA levels further emphasizes vitamin C’s role in alleviating oxidative stress, which is essential for reducing inflammation and preventing the development or progression of oxidative stress-related conditions, thereby contributing to tissue integrity and long-term protection against chronic diseases linked to oxidative mechanisms.

However, the anti-inflammatory effects of vitamin C may not fully explain its impact in all contexts. For instance, Warjukar et al. conducted a clinical study in post-MI patients, showing that despite lower baseline vitamin C levels, oral supplementation did not result in significant reductions in CRP and IL-6 levels [65]. These inconsistent findings may stem from variations in baseline vitamin C levels and dosing regimens, as individuals with lower baseline levels may respond differently to supplementation compared to those with sufficient levels. This highlights the importance of considering baseline vitamin C status when evaluating anti-inflammatory outcomes

Genetic and epigenetic differences among individuals play a crucial role in modulating the molecular impact of vitamin C on inflammatory processes. A review by Ashor et al. concluded that the anti-inflammatory effects of vitamin C were not consistently observed across multiple trials, with reductions in CRP, TNF-α, and IL-6 varying significantly [66]. Factors such as individual health status, lifestyle factors diet, and the presence of comorbidities further complicate the interpretation of vitamin C’s anti-inflammation efficacy as comprehensively revised in Table 2.

Despite the abundance of relevant data on the beneficial effects on inflammation, its role at molecular and cellular levels needs to be investigated in depth.

### 3.2. Endothelial Protection and Nitric Oxide Regulation

The dysfunction of the endothelium is a key factor in the development and progression of cardiovascular diseases. The endothelium is a critical regulator of vascular homeostasis, involved in modulating blood flow [68], coagulation [69], and immune function [70]. Eazaz Lbban et al. highlighted that maintaining endothelial function may be supported not only by pharmacological treatments but also by nutritional supplementation with strong antioxidants, like vitamin C [71]. Vitamin C sustains the optimal endothelial function through multiple mechanisms, which have already been demonstrated.

#### 3.2.1. Transcriptional Control of Oxidative Stress and Inflammation

NADPH oxidase is a major enzyme involved in the generation of ROS, which plays a critical role in regulating endothelial function and vascular health. Abnormal activity of NADPH oxidase has been closely linked to the development of various CVDs [72]. Through its antioxidant action, vitamin C inhibits NADPH oxidase activity and reduces ROS production, preserving endothelial function.

**Figure 3:**
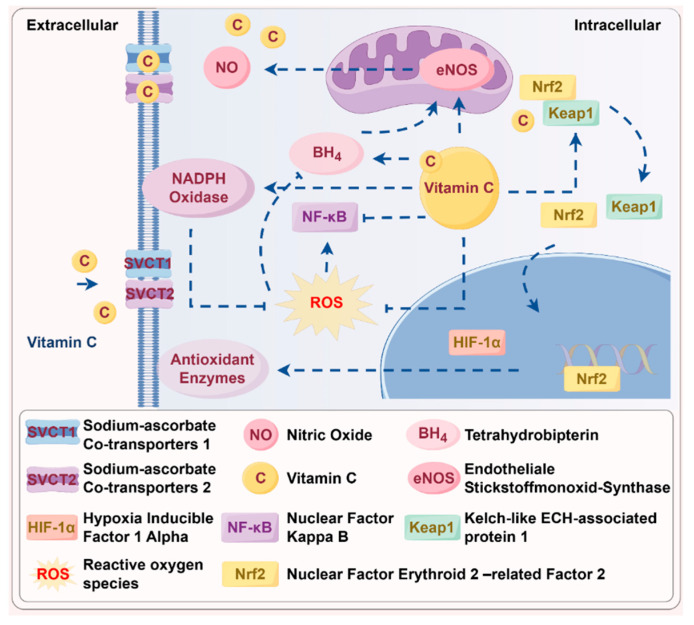
Mechanistic illustration of vitamin C-regulated signaling in endothelial cells. Vitamin C attenuates oxidative stress by inhibiting NADPH oxidase and reducing reactive oxygen species (ROS) generation. It activates antioxidant pathways through Nrf2, while suppressing pro-inflammatory NF-κB signaling. It also facilitates eNOS activation by regenerating tetrahydrobiopterin (BH_4_), enhancing nitric oxide (NO) bioavailability. Furthermore, vitamin C promotes HIF-1α degradation via prolyl hydroxylase (PHD)-mediated hydroxylation and disrupts Keap1-Nrf2 binding, enhancing antioxidant gene transcription. **Abbreviations:** ROS: reactive oxygen species; eNOS: endothelial nitric oxide synthase; NO: nitric oxide; Nrf2: nuclear factor erythroid 2-related factor 2; NF-κB: nuclear factor-κB; BH_4_: tetrahydrobiopterin; HIF-1α: hypoxia-inducible factor-1α; PHD: prolyl hydroxylase.

One of the mechanisms by which vitamin C exerts its protective effects is through the regulation of the Nrf2 and NF-κB signaling pathways. Nrf2 is a key transcription factor that promotes the expression of antioxidant enzymes, while NF-κB is involved in the inflammatory response [73]. By modulating these pathways, vitamin C downregulates NADPH oxidase expression, thereby reducing ROS production and protecting endothelial cells from oxidative and inflammatory injury. It also prevents nitric oxide (NO) synthesis from being suppressed (e.g., by preserving Endothelial Nitric Oxide Synthase (eNOS) function), thus averting endothelial dysfunction [74,75]. Furthermore, in vitro studies using HT29 intestinal epithelial cell models have demonstrated that treatment with low concentrations of vitamin C—reflecting optimal plasma levels—results in the downregulation of Cytochrome b-245 Beta Chain (CYBB), a key subunit of NADPH oxidase involved in ROS production [76]. This effect helps decrease ROS levels, mitigating oxidative stress and improving endothelial function (Figure 3).

Additionally, vitamin C has been reported to modulate the transcription of proteins crucial for endothelial function by affecting HIF-1α activity [77] and influencing histone and DNA demethylases [78]. Under normoxic conditions, vitamin C-dependent prolyl hydroxylase enzymes hydroxylate HIF-1α, marking it for proteasomal degradation. Vitamin C disrupts the interaction between Keap1 and Nrf2 by inducing structural modifications in Keap1 [79]. This process prevents inappropriate activation of HIF-1α, which otherwise would lead to the expression of genes associated with hypoxia, ensuring that the cellular responses to oxygen levels remain tightly regulated. Moreover, in a recent in vitro study, vitamin C (280 μM) was found to inhibit the aging of pulmonary vascular endothelial cells through genetically blocking the induction of Angiotensin-converting enzyme 2 (ACE2) expression in an NF-κB-dependent manner by the inflammatory factor IL-7 [80].

#### 3.2.2. eNOS Activation and Nitric Oxide Bioavailability

Vitamin C enhances the activity of eNOS, which is crucial for the production of NO, a key mediator of vasodilation and vascular homeostasis. NO bioavailability is often reduced under oxidative stress, a hallmark of endothelial dysfunction. Recently, José et al. indicated that vitamin C upregulates eNOS expression, thereby increasing NO production, which helps preserve endothelial function under adverse conditions, such as hypercholesterolemia [81]. Moreover, oxidative stress alters the phosphorylation status of eNOS by disrupting the signaling pathways in endothelial cells, thus impacting its activity [82]. For instance, vitamin C inhibits the NF-κB signaling pathway by blocking TNF-α-mediated IκB kinase (IKK) activation, reducing endothelial inflammation and preventing apoptosis in vascular smooth muscle cells [83]. Recent studies have demonstrated that vitamin C significantly improves microvascular reactivity and peripheral tissue perfusion in critically ill patients, such as those with septic shock, by enhancing NO production and promoting endothelial-dependent vasodilation, thereby mitigating microvascular dysfunction and improving tissue perfusion, even under conditions of severe endothelial impairment [84]. Lifestyle changes, such as diet and exercise, are also thought to be able to reduce oxidative stress levels, which in turn improve eNOS function [85].

Furthermore, vitamin C regenerates tetrahydrobiopterin (BH_4_), a key cofactor for eNOS. Under oxidative stress, BH_4_ is often depleted, leading to eNOS uncoupling and the sustained production of superoxide instead of NO, exacerbating endothelial dysfunction [86]. Recent studies have demonstrated that vitamin C significantly improves endothelial health by protecting BH_4_ from oxidative degradation and increasing its intracellular concentration [87]. This regenerative action of vitamin C helps improve endothelial function, reduce oxidative stress, and support cardiovascular health.

#### 3.2.3. Emerging Strategies for Endothelial Restoration

Recent advances have introduced novel therapeutic applications for vitamin C in endothelial function preservation. Combining vitamin C with dietary nitrate has been shown to further boost NO production. Vitamin C aids in the conversion of nitrate to nitrite and subsequently to NO, which enhances NO bioavailability and improves endothelial performance. This synergistic effect has been demonstrated in animal models and clinical studies, where vitamin C and nitrate supplementation together significantly reduce oxidative stress and improve vascular health [71]. This suggests a potential combination therapy for enhancing endothelial function.

Notably, early administration of a low dose of vitamin C (15 μM) in a cecal ligation and puncture (CLP)-induced septic mouse model prevented vascular endothelial damage by inhibiting BH4 oxidation, reducing oxidative stress, and improving survival [88]. Additionally, clinical evidence suggests that high-dose vitamin C (2 g orally, achieving ~50–80 μM plasma levels) can effectively restore endothelial function in inflammatory and age-related vascular conditions by enhancing NO bioavailability and reducing oxidative stress, even without significantly altering systemic inflammatory markers, like IL-6 or C-reactive protein [89].

Although in vitro studies clearly show the endothelial benefits of vitamin C, clinical results have been variable, especially regarding endothelial responsiveness and NO bioavailability. These inconsistencies suggest translational gaps between experimental models and patient outcomes. Future trials should therefore focus on endothelial-specific endpoints and mechanistic biomarkers to better define vitamin C’s clinical utility in vascular protection.

### 3.3. Fibrosis and ECM Regulation

Besides antioxidant and anti-inflammatory properties, vitamin C’s crucial role in collagen production and ECM remodeling is often underappreciated in cardiovascular therapy.

Experimental evidence has shown that vitamin C not only activates fibroblasts but also modulates key signaling pathways governing ECM remodeling [90] and tissue repair [91]. Collagen, the most abundant protein in the human body, is essential for maintaining the structural integrity of blood vessels and connective tissues. Vitamin C contributes to collagen synthesis as a cofactor for prolyl and lysyl hydroxylases [92], catalyzing the formation of hydroxyproline and hydroxylysine (post-translational modifications that stabilize the triple-helix structure of collagen and enhance its mechanical strength) [93]. These hydroxylation reactions depend on vitamin C to maintain the redox state of Fe^2+^ in enzyme active sites [94,95] and are influenced by sequence context and isoenzyme specificity [96]. Defective hydroxylation due to vitamin C deficiency leads to collagen instability, a phenomenon historically illustrated by scurvy [97]. Alarmingly, modern cases of nutritional deficiencies still occur in pediatric populations due to unbalanced dietary habits and excessive restriction of fresh produce intake [98].

These foundational mechanisms of collagen modification and ECM stabilization underlie vitamin C’s broader role in fibrotic diseases and myocardial healing, as elaborated in Chapter 4.

## 4. Therapeutic Potential of Vitamin C After Myocardial Infarction

Following MI, dramatic alterations of myocardial tissue due to the sudden loss of oxygen and nutrients force the heart to undergo critical adaptive processes. In the early stages after MI, the intense inflammatory response drives the initial activation of cardiac fibroblasts, which are influenced by upregulated proinflammatory cytokines and the influx of neutrophils [99]. As the process transitions into the late heart remodeling phase, active cardiac fibroblasts secrete ECM proteins that fill the injured area and contribute to scar formation, which is vital for maintaining cardiac structural integrity under pathological conditions [100].

### 4.1. Antioxidant and Immunomodulatory Actions in Early Post-MI Repair

Given its well-documented roles in modulating oxidative stress, inflammation, and collagen synthesis, vitamin C has been increasingly explored for its potential to improve healing and remodeling following MI. Although the clinical application of vitamin C in myocardial infarction therapy is predominantly focused on the prevention of oxidative stress and inflammation, there are solid data supporting its expanding role across all phases of healing and remodeling after MI. The potential therapeutic activity of vitamin C is dose- and time-dependent, resulting in current controversial data on its effect in heart repair, which calls for urgent and thorough re-evaluation in clinical studies.

**Figure 4:**
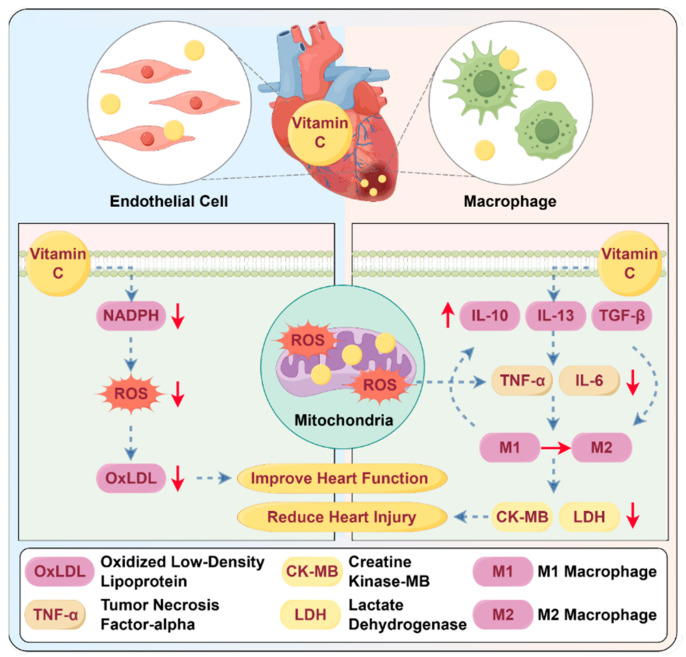
Potential role of vitamin C in early phase post-MI. Vitamin C mitigates oxidative stress and inflammation through direct effects on endothelial cells and immune modulation. In endothelial cells, it inhibits the formation of oxidized low-density lipoprotein (oxLDL), decreases ROS, and suppresses inflammatory signaling. In macrophages, it promotes polarization toward the reparative M2 phenotype, enhancing the resolution of inflammation and tissue regeneration. These combined actions preserve cardiomyocyte viability, promote cardiac repair, and facilitate the transition from inflammation to healing. Upward (↑) and downward (↓) arrows represent the upregulation or downregulation of of indicated molecules expression or activity after vitamin C supplementation. **Abbreviations:** MI: myocardial infarction; ROS: reactive oxygen species; oxLDL: oxidized low-density lipoprotein.

Reperfusion injury is characterized by the accumulation of ROS, which exacerbate damage to cardiomyocytes [101,102]. Vitamin C’s antioxidant properties can mitigate this injury, contributing to improved outcomes in the early healing process (Figure 4). Vitamin C’s cardioprotective benefits are particularly evident in its impact on key cardiac function parameters, especially in models of doxorubicin-induced cardiomyopathy (a condition where the heart’s ability to pump blood is compromised due to chemotherapy-induced toxicity) [103].

A recent meta-analysis by Harri et al., encompassing 15 trials, demonstrated that vitamin C supplementation significantly improved heart function, with the greatest benefit observed in those with lower baseline ejection fraction [104]. This protective effect is partly attributed to vitamin C’s capacity to attenuate ROS, lipid oxidation (e.g., cell membranes) [105], protein degradation (mitochondrial ferritin, MFRT, VEGF) [106,107], and DNA damage [108]. These antioxidant actions are particularly beneficial in settings, like cardiac surgery involving ischemia-reperfusion, where vitamin C administration has shown to improve patient outcomes [109].

Furthermore, vitamin C’s antioxidant properties not only protect against tissue damage but also create conditions that enable anti-inflammatory cells to act in a controlled and effective way to promote healing in the post-MI phase. The balance between pro- and anti-inflammatory signals is critical for the timely transition from the inflammatory phase to the reparative phase of heart tissue [110,111]. Vitamin C stimulates the secretion of IL-10, IL-13, and TGF-β by T regulatory cells, while simultaneously decreasing the production of pro-inflammatory cytokines, such as TNF-α, IL-6, and IL-1β [112,113]. Anti-inflammatory cytokines, including IL-10 [114], IL-13 [115], and TGF-β [116], play pivotal roles in facilitating the transition to tissue repair. These cytokines promote critical processes, such as collagen synthesis, scar formation, and angiogenesis, which are essential for effective tissue regeneration and the maintenance of cardiac function. In addition, vitamin C modulates the inflammatory reaction in the post-MI inflammatory phase by downregulating the activity of CD11b/CD18 integrins on monocytes and neutrophils expressed on the surface of monocytes and neutrophils, which consequently reduces their migration and adhesion to sites of inflammation [117,118].

Furthermore, vitamin C (at a concentration of 3 mg/mL) helps shift macrophage polarization from the pro-inflammatory M1 phenotype to the reparative M2 phenotype (as shown in vitro), which is important in controlling inflammation and promoting tissue healing [119]. Vitamin C also enhances the differentiation, maturation, and proliferation of T cells, including CD4^+^ T cell and T regulatory cells [112], thereby modulating inflammation and preventing excessive damage to cardiac tissues [113] (Figure 4).

### 4.2. Endothelial Stabilization and ECM Remodeling

In addition to systemic effects, vitamin C directly modulates endothelial integrity during the early remodeling phase post-MI. The regulation of endothelial cell function by inflammatory factors plays a crucial role in the early heart remodeling phase post-MI. Vitamin C can scavenge free radicals early in the process, preventing the formation of oxidized LDL [120]. This, in turn, reduces the adhesion of monocytes to a confluent monolayer of primary human umbilical vein endothelial cells (HUVECs), which is a key step in the initiation of inflammatory reactions [121]. Moreover, vitamin C’s ability to modulate transcription factor pathways to reduce inflammatory cytokine gene expression is dose-dependent. For example, supplementing HUVEC or ECV304 endothelial cells with 1.0–3.0 µg/mL vitamin C in vitro significantly downregulates TNF-α-driven IKK activation, a crucial step in IL-1- and IFN-γ-mediated inflammatory responses [122,123]. Additionally, vitamin C has been shown to mediate the downregulation of nuclear factor-κB (NF-κB)-related inflammation, likely through the p38 MAPK pathway [124,125].

The transition from inflammation to repair is crucial for post-MI cardiac preservation. This reparative phase is characterized by fibroblast-driven ECM production, including collagen synthesis and scar formation. Vitamin C exerts multifaceted effects during this phase by modulating fibroblast behavior, collagen homeostasis, and matrix turnover [126,127]. During the reparative phase following MI, vitamin C facilitates fibroblast activation and differentiation into myofibroblasts, a key step in post-injury ECM reconstruction [54]. This response involves both canonical TGF-β signaling [128] and alternative, Smad-independent pathways, such as those mediated by DDR1 and CCN2 in human dermal fibroblast cell lines [129]. Vitamin C not only upregulates type I and III collagen gene expression under hypoxic conditions but also exerts antifibrotic activity by suppressing TGF-β in macrophages and reducing ROS-driven signaling [130]. These multi-pathway regulatory effects position vitamin C as a modulator of both collagen deposition and inflammation-mediated fibrosis.

Beyond its direct role in collagen biosynthesis, vitamin C contributes to ECM homeostasis by regulating MMPs, which are involved in collagen degradation [131]. It enhances collagen’s resistance to enzymatic breakdown while simultaneously downregulating matrix metalloproteinases (*MMPs*) gene expression, promoting ECM stability in both physiological and pathological states, as summarized in Table 3 [132,133,134]. In hypoxic environments, such as infarcted myocardium, vitamin C enhances the activity of prolyl hydroxylases (PHDs), which hydroxylate HIF-1α and promote its degradation [135]. This protein-level regulation of HIF-1α facilitates balanced collagen gene expression without altering transcriptional machinery, ensuring appropriate ECM remodeling during tissue repair [136]. Together, these findings highlight vitamin C’s multifaceted role as a stabilizer of collagen structure, regulator of fibroblast behavior, and protector against maladaptive fibrosis.

### 4.3. Clinical Outcomes and Sex-Specific Responses

Under profibrotic conditions, vitamin C plays a crucial role in stabilizing the synthesis of de novo collagen fibrils. By enhancing the hydroxylation of proline and lysine residues, vitamin C strengthens the structural integrity of collagen, particularly types I and III, which are essential for the formation of a stable and mature scar in the infarcted area [141]. This stabilization ensures that the newly formed scar tissue is resilient enough to withstand the mechanical stresses placed on the heart post-infarction, thus preventing adverse remodeling and reducing the risk of cardiac rupture. These biochemical insights lay the groundwork for understanding observed sex-related differences in clinical outcomes, as discussed below

Despite promising preclinical evidence, clinical trials examining the role of vitamin C in late heart remodeling and MI outcomes have yielded mixed results. From a clinical point of view, the synergistic effects of vitamin C are summarized in Table 4.

A 16-year study involving 85,118 females’ nurses found that participants in the highest quintile of total vitamin C intake (≥360 mg/day from supplements and diet) had a significantly reduced risk of nonfatal and fatal MI (RR = 0.72; 95% CI 0.61 to 0.86). However, when considering dietary vitamin C alone, the association was not significant (RR = 0.86; 95% CI 0.59 to 1.26) [147]. This finding suggests potential cardiovascular protective effects of supplemental vitamin C intake in females. In contrast, other large-scale studies have shown no significant benefit of vitamin C supplementation in reducing MI incidence or improving outcomes in late cardiac remodeling. For instance, in a cohort of 14,641 American male physicians aged 50 and above, daily supplementation of 500 mg of vitamin C did not significantly decrease MI incidence (HR = 0.90; 95% CI 0.75–1.07) over 8 years [148]. Similarly, a study of females aged 40 and older with existing CVD or risk factors reported no effect of 500 mg/day of vitamin C on MI outcomes [149].

These inconsistent results suggest that physiological differences between males and females may influence responses to vitamin C supplementation. Males typically have a higher fat-free mass, resulting in a larger distribution volume and consequently lower plasma vitamin C concentrations, possibly necessitating higher supplement doses to achieve protective levels [150]. On the other hand, females might inherently exhibit enhanced antioxidant and anti-inflammatory responses due to estrogenic influences [151].

Furthermore, the differing efficacy observed between generally healthy female populations and high-risk female populations suggests that baseline cardiovascular risk may play a critical role in determining vitamin C’s cardioprotective effects [15]. These observations emphasize the need for future research to incorporate explicitly sex-specific analyses and stratification by baseline cardiovascular risk profiles. Such approaches will better delineate differential physiological responses and guide optimized, personalized supplementation strategies for preventing cardiovascular diseases for both sexes.

### 4.4. Dosing Strategies, Administration Routes, and Therapeutic Timing

The therapeutic efficacy of vitamin C in late-stage cardiac remodeling may hinge on achieving an optimal dosage range. Evidence indicates that plasma vitamin C concentrations in the range of 40–65 µM (0.007–0.011 g/L), typically achieved through oral intake of 100–200 mg/day, are considered beneficial for cardiovascular health because they support antioxidant defenses and endothelial function [36]. Higher levels, such as 70 µM (0.0123 g/L), are seldom exceeded by oral supplementation alone [38]. In contrast, intravenous vitamin C (IVC) can raise plasma levels to 1–10 mM (0.176–1.76 g/L), depending on the dosage and infusion rate [152]. Studies using IVC at 3–6 g/day or up to 12 g/day in critically ill patients, including those with heart failure or COVID-19, have reported improved cardiac outcomes and reduced inflammation [153].

However, plasma concentrations exceeding ~5 mM (0.88 g/mL) can paradoxically induce oxidative stress in erythrocytes and promote a procoagulant state via phosphatidylserine exposure and microvesicle release [154]. This procoagulant effect has been associated with excessive hydrogen peroxide generation and oxidation of red blood cell peroxiredoxin-2 (Prx2) under millimolar vitamin C conditions [155]. These findings highlight the importance of tailoring vitamin C administration by dose, route, and patient condition to balance therapeutic effects and potential risks.

Therefore, the cardioprotective benefits of vitamin C appear to be highly time-sensitive, with early post-MI administration likely yielding the greatest effect.”. Given its dual role in modulating inflammation and fibrosis, vitamin C may be particularly beneficial during the proliferative phase, which is characterized by rapid tissue turnover and ECM deposition. Future studies are needed to better define the optimal timing and dosing strategies for vitamin C supplementation to enhance outcomes in late-phase cardiac remodeling.

## 5. Considerations for Vitamin C Supplementation

### 5.1. Advanced Formulations, Production Technologies, and Delivery Systems

Advanced formulations of vitamin C, such as nano-liposomal and microencapsulated preparations, further improve absorption and prolong its bioavailability in the circulatory system. Liposomal encapsulation addresses the challenges related to the rapid breakdown and low bioavailability of free vitamin C, particularly in physiological environments where it is prone to oxidation. For instance, liposomal vitamin C can cross cellular membranes more effectively via endocytosis, enhancing its therapeutic efficacy [156,157].

Innovative biomaterials have been engineered to optimize the delivery of vitamin C in cardiovascular disease management. Electrospun vitamin C-enriched polycaprolactone fibers have shown dose-dependent ROS scavenging effects [158], while vitamin C-enriched chitosan hydrogels have demonstrated superior cardiomyocyte survival and reduced oxidative stress [159]. Such drug-loaded biomaterials offer improved stability, biocompatibility, and controlled release kinetics for vitamin C [160,161]. Recent advancements in nanotechnology have demonstrated that encapsulating vitamin C within liposomes using high-pressure homogenization (HPH) significantly enhances its stability, bioavailability, and controlled release [162]. This method ensures that vitamin C remains protected from degradation, increases its absorption efficiency, and allows for a sustained release profile, making it a promising candidate for clinical applications and large-scale production in nutraceutical industry. 

Since dietary intake of vitamin C is essential for health, the growing demand for vitamin C has driven advances in its industrial production. Besides extraction from natural sources (which is difficult to scale up reproducibly), a one-step fermentation process using *Saccharomyces cerevisiae* has been developed [163]. However, vitamin C produced by this method has primarily been applied in studies focusing on ROS scavenging and immune regulation, without exploring its additional biological roles in inflammation, angiogenesis, ECM remodeling, and fibrosis. Therefore, further research is needed to optimize vitamin C production methods to meet the metabolic complexities of its diverse biological activities.

### 5.2. Personalized Dosing and Metabolic Comorbidity Considerations

Traditional therapies for managing lipid levels, such as statins and other lipid-lowering medications, have proven effective for many individuals, but not all patients respond equally to these treatments [164,165]. Emerging evidence suggests that vitamin C may contribute to lipid regulation, offering potential as an adjunct in dyslipidemia and metabolic syndrome. An in vitro study has shown that vitamin C can help lower levels of low-density lipoprotein (LDL) cholesterol, often referred to as “bad cholesterol”, reducing its oxidation and the risk of atherosclerosis [120]. A recent observational meta-analysis suggested that vitamin C supplementation may reduce elevated triglyceride levels in individuals with metabolic syndrome or obesity, potentially through improvements in insulin sensitivity and lipid metabolism [166].

While vitamin C alone may have a positive effect on lipid regulation, its potential may be even greater when used in combination with conventional treatments. Research has shown that when vitamin C is combined with statins, the lipid-lowering effect may be synergistic, leading to improved outcomes for individuals with high cholesterol [167]. This combined approach could offer a more holistic strategy for managing lipids and improving heart health, especially for patients who may not tolerate statins well or who seek a more natural adjunct to traditional therapies.

Moreover, vitamin C has been closely linked to diabetes [168], which itself is a significant risk factor for CVDs. Patients with diabetes typically exhibit increased oxidative stress and inflammation, both of which can be modulated by vitamin C [169]. Hence, the role of vitamin C in diabetic populations, particularly its potential for improving glycemic control, reducing oxidative damage, and enhancing vascular function, merits further discussion in future studies. Although evidence suggests vitamin C may help improve glycemic control, reduce oxidative damage, and enhance vascular function in diabetic patients, more research is needed to confirm these benefits.

## 6. Conclusions and Future Perspectives

Vitamin C confers cardioprotective effects through multiple molecular pathways, including Nrf2-mediated antioxidant defense, NF-κB inhibition of inflammation, eNOS-driven nitric oxide preservation, and TGF-β-regulated extracellular matrix remodeling. These mechanisms allow vitamin C to preserve endothelial integrity, attenuate oxidative and inflammatory stress, and promote myocardial repair. This pleiotropic activity underscores its potential as a multifunctional agent in cardiovascular health.

These molecular actions are substantiated by both preclinical models and clinical studies. In particular, vitamin C supplementation has been shown to enhance endothelial function under acute inflammatory conditions and improve the left ventricular ejection fraction in post-myocardial infarction patients—especially those with reduced baseline ejection fraction, as demonstrated in the meta-analysis by Hemilä et al. [104]. These findings highlight the translational potential of vitamin C from mechanistic insight to therapeutic application.

To maximize its therapeutic benefits, optimized dosing strategies and advanced delivery systems are crucial. Oral supplementation rarely elevates plasma vitamin C levels above 70 µM, whereas IVC at 3–12 g/day can achieve pharmacologic concentrations up to 1–10 mM, offering pronounced anti-inflammatory and endothelial-stabilizing effects in critically ill or cardiovascular patients. Given its wide availability and minimal side effects, vitamin C offers a low-cost, complementary approach to traditional cardiovascular treatments. Novel formulations, such as liposomal vitamin C or biomaterial-based delivery systems, may further enhance bioavailability, tissue targeting, and sustained release, thereby amplifying clinical efficacy.

In the broader context of cardiovascular prevention and management, vitamin C also aligns well with lifestyle-based interventions, emphasizing the role of diet, micronutrient balance, and oxidative stress control. As our understanding of nutraceutical and pharmacological synergies expands, vitamin C is poised to become an increasingly important component of integrative cardiovascular care. Future large-scale clinical trials and translational studies will be essential to fully establish its role and to integrate vitamin C into evidence-based cardiovascular guidelines.

## Figures and Tables

**Table 1:** Development of cardiovascular pathology in vitamin C deficient (*GULO*^-/-^) mice.

Model	Pathophysiology	Development	Reference
*GULO*^-/-^ C57BL6 mice	Heart structural changes Cardiac dysfunction Severe emphysema TNF-α↑; MMP-2↑; MMP-9 ↑	Extensive cardiac injury leading to sudden stress death	[25]
	Left ventricular dilation ↑ Diastolic dysfunction ↑ Cardiac function ↓	100% died within 5 days following myocardial infarction	[23]
*GULO*^-/-^ C57BL/6 J mice	Heartbeat ↓ Cardiac output ↓ MMP2↑; MMP9↑; TNF-α ↑	100% died within 2 weeks following myocardial infarction	[26]
*GULO*^-/-^ BALB/cBy mice	High-density lipoprotein ↓ Low-density lipoprotein ↑	Arteriosclerosis	[24]
*GULO*^-/-^ C57BL6 mice	Antioxidative capacity ↓ High-density lipoprotein ↓ Cholesterol ↑ Vascular integrity ↓	Aneurysm, endothelial disfunction, and atherosclerosis	[27]

The upward (↑) and downward (↓) arrows indicate the upregulation or downregulation of various effectors involved in the progression of CVD in vitamin C-deficient (*GULO^-/-^*) mice. These markers highlight the physiological changes associated with vitamin C deficiency and its impact on cardiovascular pathology, providing insight into how the lack of vitamin C affects disease mechanisms.

**Table 2:** The role of vitamin C on anti-inflammation effect in clinic trials.

Study Design	Sample Size	Inflammatory Markers and Levels	Serum Vitamin C Levels	Reference
Comparative Cross-Sectional	150 (75 patients, 75 controls)	CRP: myocardial infarction patients: 4.3 ± 1.2 mg/L, controls: 2.1 ± 0.7 mg/L (no significant) IL-6: myocardial infarction patients: 7.8 ± 2.3 pg/mL, controls: 4.5 ± 1.5 pg/mL (no significant)	MI patients: <1.93732 mg/L, Controls: 1.93732–4.05076 mg/L	[65]
Secondary Analysis Cross-Sectional	7607 participants	CRP: high vitamin C: 1.4 ± 0.5 mg/L, low vitamin C: 3.2 ± 1.1 mg/L RDW (no significant)	High vitamin C levels: >12.3284 mg/L, Low vitamin C levels: <4.05076 mg/L	[67]
Clinical Trials Review	10 systematic reviews (6409 participants)	CRP: ranged from no significant change to a reduction of 1.0–2.5 mg/L in different studies IL-6: ranged from no significant change to a decrease of 1.2–3.0 pg/mL in different studies TNF-α: ranged from no significant change to a reduction of 0.5–1.5 pg/mL in different studies	From <4.05076 mg/L to >12.3284 mg/L	[66]

**Table 3:** The role of vitamin C in animal models of cardiovascular disease.

Type of Study	Vitamin C Levels	Type of MMPs	Reference
Development of living valve substitutes using porcine aortic valve interstitial cells encapsulated in PEG hydrogels	Vitamin C supplemented in the medium	MMP-2 ↑	[137]
Biomimetic approach using porcine valvular interstitial cells in 3D microtissues	250 μM (0.044 g/L) ascorbic acid 2-phosphate supplementations	MMP-1/2/3/9 ↑	[138]
HUVECs	100 µM (0.0176 g/L) vitamin C	MMP-2 ↑	[139]
Cardiac fibroblasts	Vitamin C; Arphamenine A	MMP-1/2 ↓	[140]

Upward (↑) and downward (↓) arrows represent the upregulation or downregulation of various MMPs in cellular models after vitamin C supplementation. These indicators emphasize the role of vitamin C in modulating MMP activity during ECM synthesis.

**Table 4:** Outcomes of vitamin C supplementation in clinical trials for cardiovascular diseases.

Patient and Patient Stratification	Vitamin C Levels	Study Size	Disease Development	Clinical Parameters and Significance Level	Reference
Patients after elective PCI (Vitamin C group/Placebo)	Total 1 g, 16.6 mg/min	Before/after PCI, 56 participants	Microcirculatory reperfusion improved	8-hydroxy-2-deoxyguanosine ↓↓ 8-iso-prostaglandin F2alpha ↓	[142]
Male patients aged < 65 years old (MI patients/Control group)	3.43 ± 0.21 mg/L 6.51 ± 0.32 mg/L	2.5 years, 357 participants	-	Cholesterol ↑↑ HDL-cholesterol ↓↓↓	[143]
50–70 years old patients with high coronary calcium scores (≥80%)	1 g/day	4.3 years, 1005 participants	Did not reduce non-fatal MI or coronary death	-	[144]
First-diagnosed MI (High/low levels of plasma vitamin C)	>176.12 mg/L <176.12 mg/L	84 days, 86 participants	Decrease of left ventricular ejection fraction	-	[145]
Participants without non-fatal MI (With/without vitamin C deficiency)	<2.01 mg/L >2.01 mg/L	5 years, 1605 participants	non-fatal MI	-	[146]

The upward (↑) and downward (↓) arrows in the table indicate changes in clinical parameters associated with vitamin C levels and supplementation in cardiovascular patient groups. * PCI, percutaneous coronary intervention; MI, myocardial infarction.

## Abbreviations

CVDs	Cardiovascular diseases
MI	Myocardial infarction
AS	Atherosclerosis
ECM	Extracellular matrix
ROS	Reactive oxygen species
GULO	L-gulono-1,4-lactone oxidase
RGN	Regucalcin
SVCT	Vitamin C transporters
Na+	Sodium ions
NADPH	Nicotinamide adenine dinucleotide phosphate
IL-6	Interleukin-6
TNF-α	Tumor necrosis factor-alpha
CRP	C-reactive protein
GSH-Px	Glutathione peroxidase
HPH	High-pressure homogenization
hs-CRP	High-sensitivity C-reactive protein
MDA	Malondialdehyde
NO	Nitric oxide
CYBB	Cytochrome b-245 beta chain
HIF-1α	Hypoxia-inducible factor-1α
eNOS	Endothelial nitric oxide synthase
IKK	IκB kinase
BH4	Tetrahydrobiopterin
CLP	Cecal ligation and puncture
LDL	Low-density lipoprotein
C-P4Hs	Collagen prolyl 4-hydroxylases
MMPs	Matrix metalloproteinases
PHDs	Prolyl hydroxylases
oxLDL	Oxidized LDL
HUVECs	Human umbilical vein endothelial cells
NF-κB	Nuclear factor-κB
IVC	Intravenous vitamin C
